# Incidence trends and patterns of breast cancer in Sri Lanka: an analysis of the national cancer database

**DOI:** 10.1186/s12885-018-4408-4

**Published:** 2018-04-27

**Authors:** Ashan Fernando, Umesh Jayarajah, Saumyakala Prabashani, Eshani A. Fernando, Sanjeewa A. Seneviratne

**Affiliations:** 1 Department of Surgery, Faculty of Medicine, University of Colombo, Kynsey Road, Colombo, 08 Sri Lanka; 2 National Cancer Control Programme, Ministry of Health, Colombo, Sri Lanka

**Keywords:** Breast cancer, Incidence

## Abstract

**Background:**

A gradual decline in the incidence of breast cancer is documented in developed countries especially over last two decades, while in developing countries the incidence continues to rise. We conducted this study to examine trends in incidence of breast cancer in a developing country, Sri Lanka.

**Methods:**

A retrospective cohort evaluation of patients with breast cancer during 2001–2010 was performed using population based data from the Sri Lanka National Cancer Registry. Trends in incidence were analysed using Joinpoint regression analysis.

**Results:**

The age standardized incidence of female breast cancer in Sri Lanka appears to have increased from 17.3 per 100,000 in 2001 (95% confidence interval [95% CI] 16.5–18.2) to 24.7 per 100,000 in 2010 (95% CI 23.7–25.7); a 1.4-fold increase (*p* < 0.05) with an estimated annual percentage change (EAPC) of 4.4 (95% CI 3.3–5.5). Highest incidence of breast cancer was seen among women of 60 to 64-year age group which has increased from 68.1 to 100.2 per 100,000 over this period (EAPC 4.6%, 95% CI 3.9–5.2, *p* < 0.001 for trend). A substantially greater increase was observed among women older than 50 years (from 50.4 to 76.9 per 100,000; EAPC 5.5, 95% CI 4.1–7.0, *p* < 0.05) compared with women younger than 50 years (from 32.0 to 39.6 per 100,000; EAPC 2.3, 95% CI 1.1–3.5, *p* < 0.05).

**Conclusions:**

A gradual but a significant increase in the incidence of female breast cancer is observed in Sri Lanka. A rapid rise in the breast cancer incidence among post-menopausal women appears to be the major contributor towards this increase. Improving cancer data collection appears to have been a contributor to the observed increase. However, an inherent increase is also likely as differential rates of increase were observed by age groups. Further research is needed to identify the reasons for the observed increase which may help with future cancer control efforts in Sri Lanka.

## Background

Breast cancer is the most common cancer affecting women worldwide [1]. Over the last several decades, the incidence of breast cancer has risen globally [2, 3] and this is estimated to increase by another 25% by year 2020 [4]. Studies have shown that the greatest increase will be among women in developing countries, a majority of whom live in the Asian region [4, 5]. In India alone, over 100,000 new cases of breast cancer are estimated to be diagnosed annually [6]. According to data from the International Agency for Research on Cancer (IARC), a disproportionately high number of breast cancer deaths occur in developing countries due to lower cancer specific survival rates [4]. Most studies on incidence and trends in incidence of breast cancer have been conducted in developed countries, while analyses of these trends and patterns in developing countries including Sri Lanka are limited.

Mammographic breast cancer screening has contributed significantly towards the reduction in breast cancer mortality observed in developed countries where population based screening programmes are in operation [7]. However, the same has contributed to an increase in the incidence of breast cancer as many cancers which otherwise would not have manifested clinically, are diagnosed through screening [8]. Many developing countries including Sri Lanka does not have a national breast cancer screening programme. This probably is one reason for the higher proportion of advanced breast cancers at diagnosis observed in Sri Lanka, compared with developed countries [9]. On the other hand, lack of a national screening programme has likely contributed to the lower breast cancer incidence in Sri Lanka compared with the developed countries.

Since 1985, Sri Lanka National Cancer Control Programme (NCCP) has been collecting nationwide cancer data. Over this period, the coverage has gradually increased, and as of 2014 it is estimated to include over 80% of all cancers diagnosed in the country [10, 11]. NCCP data include all cancers treated at national cancer treatment centres and data from other major private and government hospitals, and pathology laboratories. As adjuvant treatments in the public sector are instituted almost exclusively through national cancer treatment centres, the overall coverage for breast cancer is likely to be greater than 80%.

This study was conducted with the aim of identifying recent trends in breast incidence Sri Lanka, and to compare these tends with other countries.

## Methods

Details of all patients with newly diagnosed primary breast cancer between 01/01/2001 and 31/12/2010 were extracted from cancer incidence data of Sri Lanka published by the NCCP [12].

Age standardized rates of breast cancer per 100,000 population were calculated for each year using World Health Organization (WHO) age standardized populations [13]. Similar methods were used to calculate age group specific and gender specific incidence rates of breast cancer for each year under consideration.

Significance of the trends in incidence and annual changes in incidence rates were analysed using Joinpoint software [14]. Joinpoint regression analysis identifies points where a statistically significant change over time in linear slope of the trend occurred. This analysis starts with the minimum number of joinpoints, and tests whether one or more joinpoints are statistically significant, and are added to the model. Joinpoint tests of significance use a Monte Carlo permutation method [15]. In the final model, each joinpoint indicates a statistically significant change in trend, and an estimated annual percentage change (EAPC) computed for each of those trends by means of generalized linear models assuming a Poisson distribution. Significance of the change in rates were calculated with *p* values and *p* values < 0.05 were considered as statistically significant. Joinpoint software version 4.3 was used for Joinpoint regression analysis [14].

## Results

This study analysed a total of 19,755 patients with newly diagnosed primary invasive breast cancer over the 10-year study period. Approximately 98% (*n* = 19,291) were females and 2% (*n* = 464) were males. The female to male ratio was 41.7: 1.

**Fig. 1:**
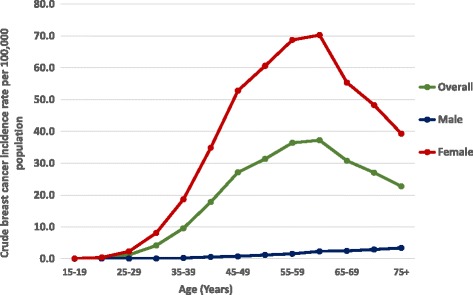
Crude breast cancer incidence rates by age group in Sri Lanka, 2001–2010

The overall mean age of the patients was 53.1 years (median 52 years). Males were significantly older compared with females at diagnosis (males 58.7 vs. females 52.9 years, *p* < 0.001). Invasive ductal carcinoma was the commonest reported histological type (79.3%, *n* = 15,341) followed by lobular carcinoma in 3.6% (*n* = 721). Rare variants of breast cancer were reported in 5.7% (*n* = 1124) patients, while specific histological variant was not documented in 11.4% (*n* = 2244) patients. The highest incidence of female breast cancer was seen in 60–64 age group (70.3 per 100,000 population), while the highest incidence of male breast cancer was seen in over 75+ year age group (3.38 per 100,000 population) (Fig. 1).

**Fig. 2:**
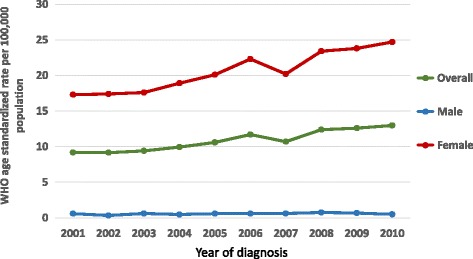
WHO age standardized incidence rates for breast cancer per 100,000 population in Sri Lanka 2001–2010

The WHO age standardized incidence of breast cancer in Sri Lanka was observed to have increased from 9.2 per 100,000 in 2001 (95% confidence interval [95% CI] 8.17–9.62) to 12.9 per 100,000 in 2010 (95% CI 12.4–13.5); a 1.4-fold increase, (*p* < 0.05 for trend) (Fig. 2). This increase translates into an estimated annual percentage change (EAPC) in incidence of 4.4 (95% CI 3.3–5.4) over this 10-year period. The EAPC for females was 4.4 (95% CI 3.3–5.5, *p* < 0.001 for trend) and for males it was 3.1 (95% CI -2.5-9.1, *p* > 0.05 for trend).

**Fig. 3:**
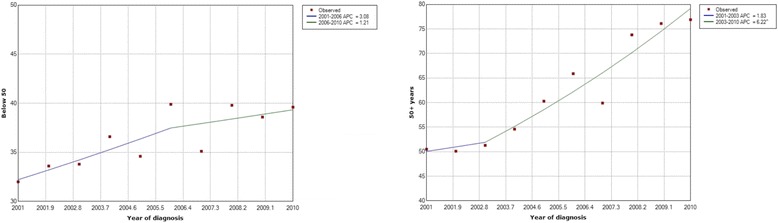
Joint point trend graphs for breast cancer incidence by age below and above 50 years at diagnosis of breast cancer in Sri Lanka 2001–2010

Results of the Joinpoint analysis of incidence time trends by age, gender and histology type are shown in Table 1. The EAPC for overall female age standardized incidence rate was 4.4 (95% CI 3.3–5.5) which was similar to the overall EAPC rate. A greater part of the increase in breast cancer incidence is attributable to an increase in incidence of ductal carcinoma which has increased from 7.2 to 11.3 per 100,000; a 1.56-fold increase (EAPC = 5.2, 95% CI 2.8–7.8, *p* < 0.05 for trend) (Table 1). In comparison, the increase in incidence of lobular carcinoma was observed to be minimal (EAPC = 2.6, *p* > 0.05). Progressively increasing EAPCs were observed with advancing age. EAPC was significantly higher among women older than 60 years (EAPC = 5.55) compared with women of 40–59 (EAPC = 3.09) and 20–39 (EAPC = 2.12) year age groups. Furthermore, only a modest increase in the incidence of breast cancer was observed among women younger than 50 years (from 32.0 to 39.6 per 100,000; EAPC = 2.3, 95% CI 1.1–3.5, *p* < 0.05), while the increase was substantially greater among women older than 50 years (from 50.4 to 76.9 per 100,000; EAPC = 5.5, 95% CI 4.1–7.0, *p* < 0.05) (Fig. 3).

**Table 1:** Breast cancer incidence in Sri Lanka by gender and age group with Joinpoint analysis of Estimated Annual Percentage Change (EAPC) from 2001 to 2010

	2001		2010		EAPC 2001–2010 (95% CI)
	n	Rate (95% CI)	n	Rate (95% CI)	
Age group (years)					
Male					
< 20	1	0.03	1	0.03	0
20–39	2	0.07	2	0.06	−1.43
40–59	15	0.72	10	0.44	−3.89
60+	26	3.16	26	2.88	−0.89
Age standardized	44	0.58 (0.41–0.76)	39	0.48 (0.33–0.63)	3.1 (−2.5–9.1)
Female					
< 20	2	0.06	2	0.06	0
20–39	197	6.42	263	7.78	2.12*
40-59	952	44.6	1374	58.4	3.09*
60+	396	48.1	755	74.8	5.55*
Age standardized	1547	17.3 (16.5–18.2)	2401	24.7 (23.7–25.7)	4.4 (3.3–5.5)*
Overall age standardised rate	1591	9.17 (8.17–9.62)	2440	13.0 (12.5–13.5)	4.4 (3.3–5.4)
Histology type					
Female					
Ductal CA	1221	13.6 (12.9–14.4)	2085	21.4 (20.5–22.4)	5.2 (2.8–7.8)*
Lobular CA	74	0.8 (0.6–1.0)	55	0.6 (0.4–0.7)	−3.3 (− 10.4–19.2)
Malignancy NOS	158	1.8 (1.5–2.1)	142	1.5 (1.2–1.7)	−0.9 (− 10.6–9.9)
Others	94	1.0 (0.8–1.3)	119	1.2 (1.0–1.5)	2.4 (−2.4–7.5)
Male					
Ductal CA	32	0.43 (0.28–0.58)	33	0.41 (0.27–0.55)	3.2 (−3.3–10.3)
Lobular CA	2	0.02 (0.00–0.06)	0	0.0	–
Malignancy NOS	4	0.05 (0.00–0.10)	3	0.04 (0.02–0.08)	5.6 (− 12.1–26.9)
Others	6	0.08 (0.02–0.14)	3	0.03 (0.00–0.07)	− 4.3 (− 20.3–15.1)
Overall					
Ductal CA	1253	7.21 (6.82–7.64)	2118	11.2 (10.8–11.7)	5.2 (2.8–7.7)*
Lobular CA	76	0.44 (0.34–0.54)	55	0.29 (0.21–0.36)	2.6 (− 10.4–17.6)
Malignancy NOS	162	0.95 (0.82–1.13)	145	0.78 (0.65–0.90)	−0.8 (− 10.8–10.3)
Others	100	0.57 (0.46–0.68)	122	0.66 (0.54–0.78)	2.8 (−2.5–8.3)

*The EAPC is significant (*p* < 0.05)

## Discussion

This study based on data from the Sri Lanka National Cancer Registry (SLCR) appears to show a steady increase in the incidence of female breast cancer. The incidence in males has remained largely static. Further, the breast cancer incidence appeared to be rising more rapidly among post compared with pre-menopausal women.

Sri Lanka has a free public health care system, although private health systems which run parallel make a substantial contribution [16]. The burden of cancer care rests almost exclusively with the public sector due to high cost of cancer care which is beyond the affordability of most average Sri Lankans [17]. Hence, studying the incidence patterns of breast cancer is important for the government to make policy decisions on allocating funds and other resources for early detection and treatment of breast cancer.

In general, there has been a gradual and a uniform increase in the incidence of breast cancer over the study period, although a slight drop in the incidence is observed in 2007 (Fig. 2). This is likely to be due to a change in criteria adopted by the NCCP for cases to be included in the cancer registry. Prior to 2007 all invasive as well as in-situ neoplasms (ductal and lobular carcinoma in-situ) were included in the registry, while from 2007 onwards only invasive neoplasms are included. Exclusion of in-situ cancers post-2007 may have contributed to an underestimation of the actual increase in breast cancer incidence.

The observed increase in incidence is likely to be due to many factors which include improvement of the coverage of the national cancer data, increase in diagnosis due to breast cancer screening and a true increase in the incidence. Coverage of SLCR has gradually increased overtime with a current coverage rate of over 80%. Number of sources through which the national cancer registry collects data has gradually increased over the study period helping to improve the proportion of cancers reported to the registry [12]. However, even between the years where the data sources to the registry have remained constant (i.e 2004–2005 and 2008–2009), an increase in the incidence could be observed [12].

At the same time, it is likely that there has been a true increase in the number of cancers diagnosed in the country. This again is due to multiplicity of reasons. First, there has been a gradual increase in mammographic screen detected breast cancer in Sri Lanka [18]. Although Sri Lanka does not have a national breast cancer screening programme, many government and private institutions have started providing opportunistic breast cancer screening especially over the last decade [19]. This has gradually increased the proportion of screen detected breast cancer which probably has contributed to the increasing incidence.

Despite all these possible reasons for an ‘artificial’ increase in the incidence, it is likely that there has been a genuine increase in the incidence similar to many other developing countries [1]. Several factors have been proposed to be possible contributors towards this increase. These include westernization of lifestyle including increased consumption of processed and fatty food, sedentary lifestyle leading to increased obesity, delay in childbearing and reduced rates and duration of breast feeding [20]. Recently concluded WHO non-communicable disease risk factor survey 2015 reported the percentage of Sri Lankan females aged 18–69 who were obese to be 8.4% compared to 5.9% in 2006 [21]. In the present study, a significantly greater increase in the incidence of breast cancer was observed among post compared with premenopausal women. This age specific increase in incidence in older women suggest the possibility of a true increase in incidence, as general registry improvements are expected to have affected all ages similarly. Furthermore, in a similar national cancer database analysis on thyroid cancers in Sri Lanka, no differences in age specific increase in incidence was not observed, which further strengthens the argument that the age specific increase in incidence in breast cancer is due to a true increase [11].

The increase in incidence of breast cancer in older women highlights the impact of obesity on rising incidence of breast cancer as rates of obesity are much greater among post than premenopausal women in Sri Lanka [21]. In addition, the percentage of females not meeting WHO recommendations on vigorous physical activity for health was 89.2% in 2015. Late age at first birth and lower fertility which are factors proven to increase the risk of development of breast cancer have also been on the rise [22]. All these have likely have contributed to increase the risk of woman developing a breast cancer and thereby towards the increasing breast cancer incidence.

Sri Lanka has one of the fastest ageing populations in the world [23]. This trend will exponentially increase the numbers of breast cancer especially among older women. Older women are more likely to have more comorbidities and a poorer survival from breast cancer [24]. On the other hand, this increase among older age groups may reflect different rates in adoption of opportunistic breast cancer screening in Sri Lanka. However, evidence from other countries suggest that participation in opportunistic mammographic screening is greater in women younger than 50 years, compared with older women [25]. Regardless, healthcare policy makers in the country need to consider all these factors in planning strategies, if they are to effectively deal with the increasing burden of breast cancer in the country.

There were several limitations in this study. First, the coverage of breast cancer data collection has increased gradually over the study period which likely has contributed to the observed increase in the breast cancer incidence. Another possible limitation of incidence data gathered from 2001 to 2010 includes the civil war in the Northern and Eastern parts of the country (1983–2009). During this period access to health care would have not been uniform in these parts of the Island and the number of breast cancers that reported may not have been accurate. Further, cancer staging data records of NCCP were not complete up to 2007, which prevented us from performing a meaningful analysis of trends in breast cancer stage at diagnosis in Sri Lanka. Analysing the trends in stage distribution would have helped to better evaluate the trends in stage at diagnosis and effectiveness of the strategies to promote early detection of breast cancer. NCCP data did not include many other important information that would have helped to identify possible reasons for the observed trends in incidence. For instance, tumour biological characteristics including receptor status, body mass index, method of detection, menopausal status and other established breast cancer risk factors were not available in the NCCP database. Collection of such information in the future may help better understand the reasons for observed increases in incidence.

## Conclusion

A gradual increase in the incidence of female breast cancer by approximately 4% per year is observed in Sri Lanka. A rapid rise in the breast cancer incidence among post–menopausal women appear to be the major contributor towards this increase. As Sri Lanka has one of the fastest ageing populations this trend is likely to increase the number of breast cancers among older women exponentially. Healthcare policy planners need to be aware of these trends to effectively tackle the increasing burden of breast cancer in the country. Future research needs to focus on identifying reasons for the observed increase to guide effective cancer control measures.

## Abbreviations

CI: Confidence interval
EAPC: Estimated annual percentage change
NCCP: National cancer control programme
WHO: World health organization
